# Biomolecular characterization of exosomes released from cancer stem cells: Possible implications for biomarker and treatment of cancer

**DOI:** 10.18632/oncotarget.2462

**Published:** 2014-10-28

**Authors:** Dhruv Kumar, Dwijendra Gupta, Sharmila Shankar, Rakesh K. Srivastava

**Affiliations:** ^1^ Department of Pharmacology, Toxicology and Therapeutics, and Medicine, The University of Kansas Medical Center, 3901 Rainbow Boulevard, Kansas City, KS, 66160, USA; ^2^ Center of Bioinformatics and Department of Biochemistry, University of Allahabad, Allahabad, India; ^3^ Kansas City VA Medical Center, 4801 Linwood Boulevard, Kansas City, MO 64128, USA

**Keywords:** CSCs, Rotenone, CD9, CD63, CD81, Alix, Atg7, LC3

## Abstract

Cancer recognized as one of the leading irrepressible health issues is contributing to increasing mortality-rate day-by-day. The tumor microenvironment is an important field of cancer to understand the detection, treatment and prevention of cancer. Recently, cancer stem cell (CSC) research has shown promising results aiming towards cancer diagnostics and treatment. Here, we found that prostate and breast cancer stem cells secreted vesicles of endosomal origin, called exosomes showed strong connection between autophagy and exosomes released from CSCs. Exosomes may serve as vesicles to communicate with neoplastic cells (autocrine and paracrine manner) and normal cells (paracrine and endocrine manner) and thereby suppress immune systems and regulate neoplastic growth, and metastasis. They can also be used as biomarkers for various cancers. We detected tetraspanin proteins (CD9, CD63, CD81), Alix and tumor susceptibility gene-101 (TSG101) of exosomal markers from rotenone treated CSCs. We have also detected the induction of autophagy genes, Atg7 and conversion of autophagy marker (LC3-I to LC3-II), and tetraspanin proteins (CD9, CD63, CD81) in rotenone treated CSCs by western blotting. The mRNA expression of CD9, CD63, CD81 and TSG101 analyzed by qRT-PCR showed that the rotenone induced the expression of CD9, CD63, CD81 and TSG101 in CSCs. Electron microscopy of rotenone treated CSCs showed the mitochondrial damage of CSCs as confirmed by the release of exosomes from CSCs. The constituents of exosomes may be useful to understand the mechanism of exosomes formation, release and function, and also serve as a useful biomarker and provide novel therapeutic strategies for the treatment and prevention of cancer.

## INTRODUCTION

Cancer is one of the serious health issues of current scenario arisen by alterations of cellular genome and affecting expression and function of oncogenes and tumor suppressor genes. It is extensively accepted that tumor microenvironment plays an essential role in tumor development and progression [1, 2]. The complexity between cancer cells and their environment makes the identification of the factors that participate in the cancer origination is very difficult. It is significant to study the mechanisms employed by cancer cells to hide from immune recognition and support their existence and progression. The ability of cancer cells to secrete small vesicles (known as exosomes or microvesicles) is also believed to contribute in a growing number of immunosuppressive and modulating functions.

Rotenone has been shown to induce autophagy in retinal pigment epithelium and release of extra cellular microvesicles and exosomes by damaging mitochondrial activity [3]. Rotenone is a naturally derived plant product mainly obtained from the pea family members. The use of rotenone by human being may be ancient, as early explorers noted peruvian natives using crude extracts of the cube plant to stun fish for eating [4, 5]. Rotenone is classified as a member of isoflavones compounds on the basis of their molecular structure. Rotenone is well known for its toxicity to the fish. The toxicity of rotenone is because of its efficacy in interrupting mitochondrial electron transport which hinders the utilization of oxygen in respiratory organisms, leading to cell death in many organisms including insects [6]. Rotenone has been shown to result in the systemic inhibition of mitochondrial complex-I activity, which leads to the degeneration of dopaminergic neurons within the substantia nigra and striatum [7, 8]. The defective mitochondria and other organelles are cleared from the cell by a process known as autophagy in which fusion of damaged material with lysosomes and digestion is the disposal pathway [3, 9].

Exosomes are small membrane vesicles of 30–100 nm in diameter resemble to the internal vesicles present in multivesicular endosomes [10, 11]. Exosomes are released from a variety of different cell types including tumor cells, red blood cells, lymphocytes, platelets, and dendritic cells [12, 13]. Exosomes have been found in various body fluids such as blood plasma [14], malignant ascites [15], and urine [16]. It has been proposed that under physiological conditions exosomes could play a role in cell-cell interactions [17, 18]. When released from cancer cells, exosomes can promote invasion and migration [19, 20]. Depending on the cellular origin, exosomes recruit various cellular proteins that can be different from the plasma membrane including major histocompatibility complex molecules, annexins, flotillin, Alix, TSG101, integrins and tetraspanins (CD9, CD37, CD53, CD63, CD81, CD82 and CD151) [21–23], and they are enriched in raft-lipids, such as cholesterol, ceramide and sphingolipids [10–13, 19]. CD9, CD63 and CD81 belong to the tetraspanin family, which are characterized by the presence of 4 hydrophobic transmembrane domains. CD9, CD63 and CD81 are expressed in many different types of cells including neural cells, cancer cells and cancer stem cell lines. TSC101 is a multi-functional tumor susceptibility gene involved in several molecular and biological processes. Tetraspanins are categorized in the presence of four conserved transmembrane regions [21–23]. They are associated with the regulation of cell-cell communication [24, 25], cell fusion [26–29], tumor cell metastasis [30–33], cell motility [30–33], cellular activation [34–36] and signal transduction [34, 35].

Indent exosomes are membrane vesicles that form within late endocytic compartments, which are called multivesicular bodies, and are secreted upon fusion of these compartments with the plasma membrane [12, 17]. Various hematopoietic and nonhematopoietic cell types secrete exosomes, including reticulocytes, B-lymphocytes, platelets, mastocytes, several tumor cells, intestinal epithelial cells and T-lymphocytes. At present, it is unclear whether exosomes have any biological function and, if so, what these functions are. The molecular composition of exosomes from different cellular sources has been analyzed. CSCs-derived exosomes contain several proteins that are potentially involved in their biogenesis, targeting and putative immunological function. Proteins that may be involved in exosome biogenesis in multivesicular bodies include TSG101, several annexins, Rab GTPases and several signal transduction molecules (e.g., 14-3-3, a heterotrimeric G protein and Alix). Exosomes also accumulate different members of the tetraspanin family, including CD9, CD63 and CD81. Although their precise function is uncertain, tetraspanins are enriched in membrane micro domains and form networks with several other membrane proteins [37–39].

The formation of exosomes is not clearly understood. A more recent view suggests that exosomes are formed by invagination and budding from the limiting membrane of late endosomes [40]. Exosomes accumulate in cytosolic multivesicular bodies from where they are released by fusion with the plasma membrane. The process of vesicle shedding is particularly active in proliferating cells, such as cancer cells, where vesicles release can occur continuously [41]. Exosomes have also been shown to transfer oncogenic signaling receptors from one cell to another, as well as a specific subset of mRNAs and microRNAs [10, 11, 42].

The purpose of this study was to investigate the release of exosomes from prostate and breast CSCs and characterize the exosomal markers released from the prostate and breast CSCs. Exosomes carry several different kinds of biomolecular informations (mRNA, microRNA, protein, etc) and spread from cell to cell. We have demonstrated that exosomes released from the prostate and breast CSCs carries important information that could help us to understand the complexity of cancer progression and its therapy.

## RESULTS

### Rotenone induced exosomes release in CSCs

**Figure 1:**
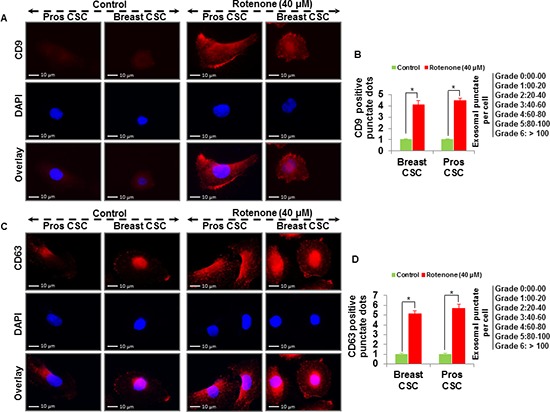
Detection of exosomal markers CD9 and CD63 in prostate and breast CSCs **(A)** Rotenone (40 μM) treated prostate and breast CSCs released CD9 marker of exosomes (scale bar, 10 μm). **(B)** Quantification of CD9 exosomes release from the prostate and breast CSCs. **(C)** Rotenone (40 μM) treated prostate and breast CSCs released CD63 marker of exosomes (scale bar, 10 μm). **(D)** Quantification of CD63 exosomes release from the prostate and breast CSCs. Data represent mean ± s. e. m. from at least three independent experiments. *P<0.05 compared with rotenone-treated and control.

**Figure 2:**
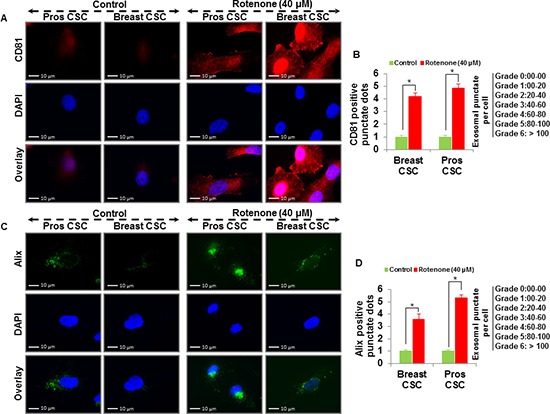
Detection of exosomal markers CD81 and Alix in prostate and breast CSCs **(A)** Rotenone (40 μM) treated prostate and breast CSCs released CD81 marker of exosomes (scale bar, 10 μm). **(B)** Quantification of CD81 exosomes release from the prostate and breast CSCs. **(C)** Rotenone (40 μM) treated prostate and breast CSCs released Alix marker of exosomes (scale bar, 10 μm). **(D)** Quantification of Alix exosomes release from the prostate and breast CSCs. Data represent mean ± s. e. m. from at least three independent experiments. *P<0.05 compared with rotenone-treated and control.

To examine whether rotenone induces release of exosomes in CSCs, we treated CSCs with rotenone (40 μM) for 48 h [3]. We analyzed the vesicles released from the human prostate and breast CSCs under fluorescence microscope. Using fluorescent antibodies against exosomal markers, CD9, CD81, CD63 and Alix, we observed the appearance of vesicles tagged with exosomal markers in CSCs treated with rotenone. The expression of exosomal markers was significantly higher in rotenone-treated CSCs compared to untreated CSCs (Figure 1 and 2). These data indicate that CSCs releases more exosomes under exposure of rotenone. Rotenone has already been shown to induce autophagy and mitochondrial damage in several organisms. Moreover, the release of exosomes could play some important role to induce autophagy in CSCs.

### Detection of exosomes and expression of exosomal markers in exosomes released from CSCs

**Figure 3:**
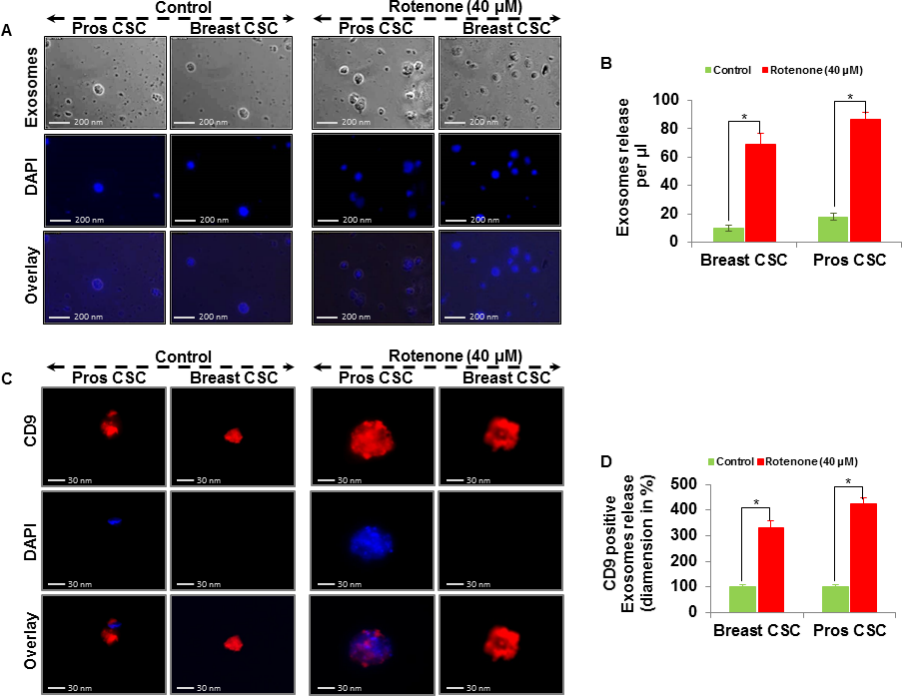
Exosomal release in rotenone treated prostate and breast CSCs **(A)** Rotenone (40 μM) treated prostate and breast CSCs released exosomes (scale bar, 200 nm). **(B)** Quantification of exosomes release from the prostate and breast CSCs. **(C)** Rotenone (40 μM) treated prostate and breast CSCs released CD9 marker of exosomes (scale bar, 30 nm). **(D)** Quantification of CD9 exosomes release from the prostate and breast CSCs. Data represent mean ± s. e. m. from at least three independent experiments. *P<0.05 compared with rotenone-treated and control.

Extracellular vesicles can be isolated from the supernatant of human CSCs by differential centrifugations. Here, we examined the exosomes release from the supernatant of human prostate and breast CSCs culture under phase contrast microscope (Figure 3A and B). The rotenone treated CSCs released exosomes more than the untreated CSCs. Furthermore, by employing fluorescent antibodies we detected CD9, CD81 and Alix in exosomes released from the CSCs under fluorescence microscope (Figure 3C and D, and 4A, B, C and D). The number and size of the exosomes released from the rotenone treated CSCs were comparatively 3-4 times larger than the exosomes released from the untreated CSCs. Also, DAPI staining of exosomes showed that the exosomes released from the CSCs contains DNA fragments. These result suggest that the exosomes released from the CSCs carries several cellular information, which could help to understand the tumor generation, progression and its metastatic properties.

**Figure 4:**
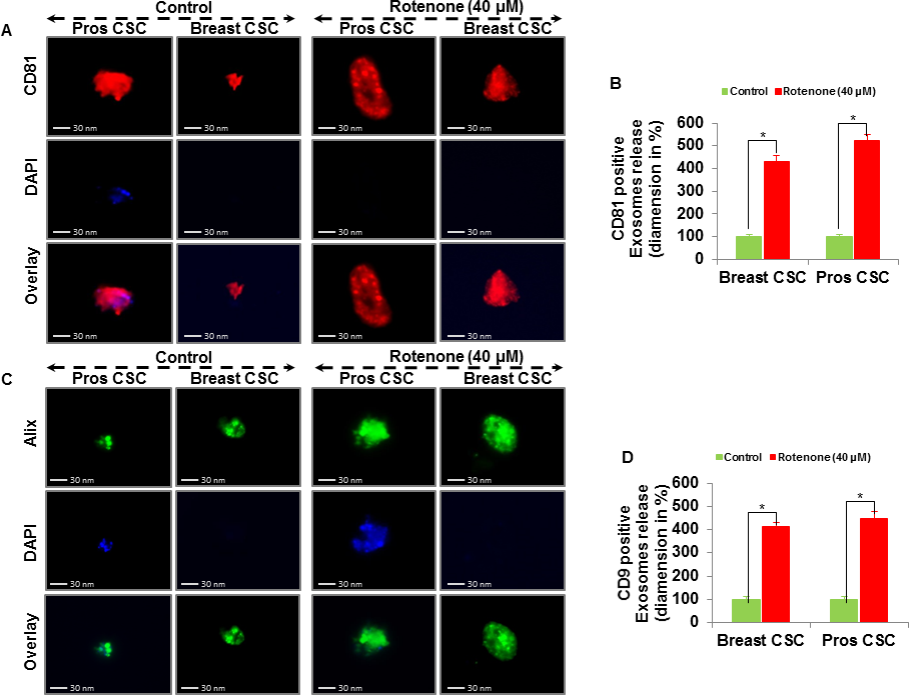
Exosomal release in rotenone treated prostate and breast CSCs **(A)** Rotenone (40 μM) treated prostate and breast CSCs released CD81 marker of exosomes (scale bar, 30 nm). **(B)** Quantification of CD81 exosomes release from the prostate and breast CSCs. **(C)** Rotenone (40 μM) treated prostate and breast CSCs released Alix marker of exosomes (scale bar, 30 nm). **(D)** Quantification of CD9 exosomes release from the prostate and breast CSCs. Data represent mean ± s. e. m. from at least three independent experiments. *P<0.05 compared with rotenone-treated and control.

### Induction of autophagy and mitochondrial damage in rotenone treated CSCs

**Figure 5:**
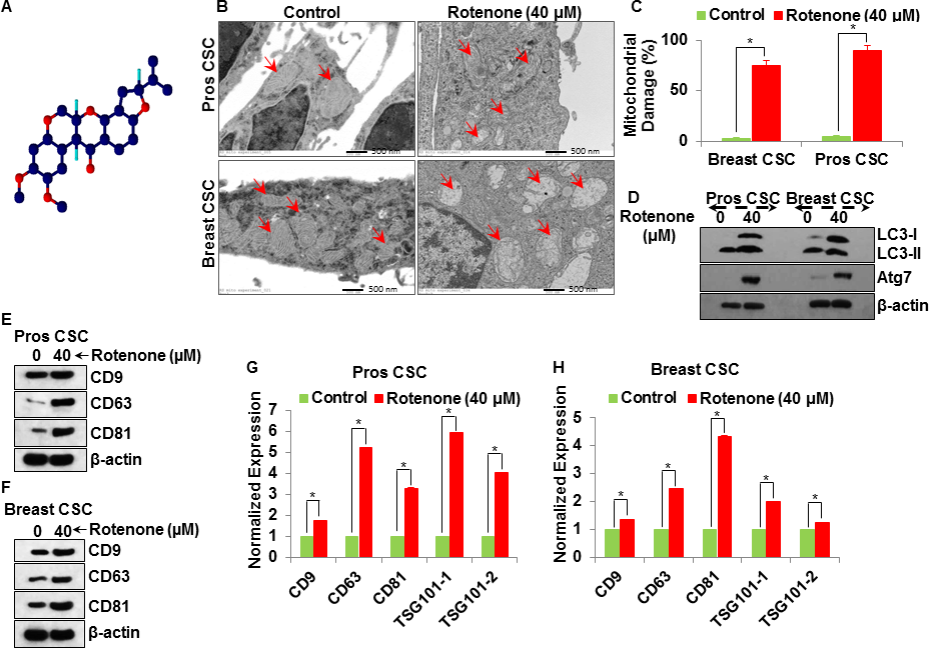
Molecular mechanism of exosomal release from the rotenone (40 μM) treated prostate and breast CSCs **(A)** Chemical structure of rotenone. **(B)** Electron microscopy of mitochondrial damaged in prostate and breast CSCs by rotenone (40 μM) (scale bar, 500 nm). **(C)** Quantification of mitochondrial damage in prostate and breast CSCs. **(D)** Activation of autophagy (LC3 and Atg7) in rotenone (40 μM)-treated prostate and breast CSCs. **(E)** Detection of exosomal markers CD9, CD63 and CD81 in exosomes isolated from rotenone-treated prostate CSCs by western blotting. **(F)** Detection of exosomal markers CD9, CD63 and CD81 in exosomes isolated from rotenone-treated breast CSCs by western blotting. **(G)** mRNA expression of exosomal markers (CD9, CD63, CD81 and TSG101) in exosomes released from the prostate CSCs. **(H)** mRNA expression of exosomal markers (CD9, CD63, CD81 and TSG101) in exosomes released from the breast CSCs. Data represent mean ± s. e. m. from at least three independent experiments. *P<0.05 compared with rotenone-treated and control.

In this experiment, we assume that the mitochondrial damage could play a significant role in the induction of autophagy and release of exsosomes in CSCs. Figure 5A shows the chemical structure of rotenone. To examine the effect of rotenone on mitochondrial damage in CSCs, we performed electron microscopy of rotenone treated (0 and 40 μM for 48 h) CSCs. Following careful fixation and preparation, damaged mitochondria were readily identified using electron microscopy (Figure 5B). Upon examination of the electron microscopy images, we found that rotenone induced extensively mitochondrial damage in CSCs (Figure 5B and C), marked by the red arrow (Right picture Figure 5B). Next, we investigated the autophagy induction in rotenone treated CSCs by western blotting. We detected expression of autophagy genes, Atg7 and conversion of autophagy marker, LC3 (LC3-I-LC3-II) by western blotting (Figure 5D). Furthermore, rotenone treated CSCs expressed more Atg7 than untreated CSCs.

We next measured the expression of tetraspanin proteins (CD9, CD63, CD81) in exosomes released by control and rotenone-treated CSCs (Figure 5E and F). We have detected the increased expression of CD9, CD63, and CD81 in exosomes released from rotenone-treated breast and prostate CSCs compared to that of untreated control. The expression of CD9, CD63 and CD81 proteins confirmed the release of exosomes by breast and prostate CSCs. These observations support the concept of association between mitochondrial damage, induction of autophagy and exosomes release in CSCs.

### mRNA expression of CD9, CD63, CD81 and TSG101 in exosome release from CSCs

Rotenone-treated CSCs released more exosomes than the untreated CSCs and the expression of CD9, CD63, CD81 and TSG101 were higher in rotenone-treated CSCs as detected by qRT-PCR (Figure 5G and H). These results suggest that the exosomes released from CSCs also carries information at mRNA level, which could be helpful to understand the cancer prevention and therapy.

## DISCUSSION

In the present study, we investigated that CD9, CD63, CD81, Alix and TSG-101 was an appropriate collection markers of exosomes derived from prostate and breast CSCs. We have demonstrated that exosomes derived from the rotenone treated prostate and breast CSCs contains several exosomal markers (CD9, CD63, CD81, Alix and TSG-101) and play a significant role in the induction of autophagy. In this study, we have characterized the exosomes released from the prostate and breast CSCs, and investigated the molecular mechanism of induction of autophagy and exosomes released from the CSCs.

**Figure 6:**
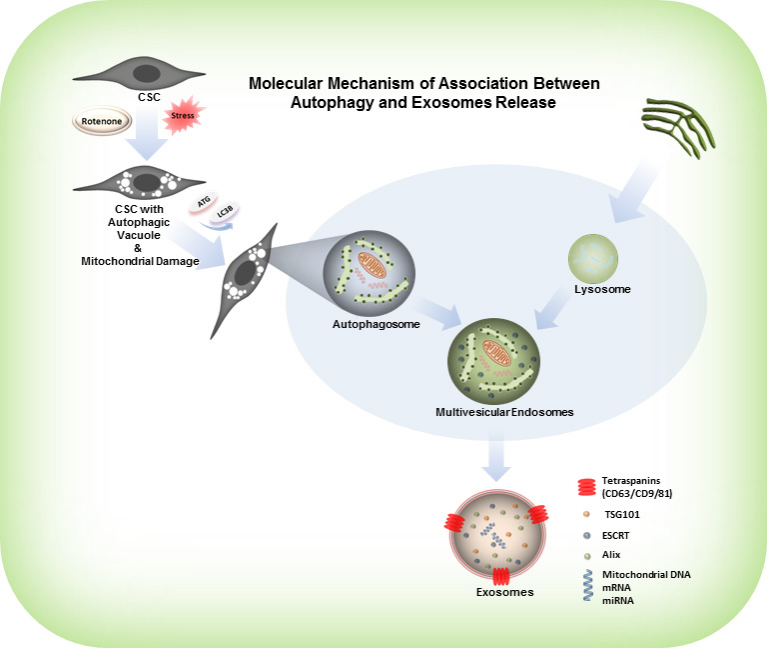
Model represents the activation of autophagy, formation of autophagosomes and release of exosomes from the rotenone treated CSCs Rotenone induces autophagic vacuolation in CSCs which are associated with the mitochondrial damage. These processes induce the formation of autophagosomes and autophagy in CSCs. Autophagosomes combine with the protein complexes, ribosomes, endoplasmic reticulum and peroxisomes, and form multi-vesicular endosomes. The multi-vesicular endosomes release exosomes.

Rotenone is a respiratory chain complex-I inhibitor, and it acts trough generation of ROS and oxidative damage of mitochondria [43, 44]. Several studies suggested that mitochondria regulate cell necrosis, apoptosis, mitochondrial dysfunction and antioxidant production through mitochondrial enzymatic activity [45]. Rotenone was shown to activate caspase-2 in mice neurons inducing the activation of downstream apoptotic effectors such as caspase 3, 9, Bid and Bax thus initiating apoptosis [46]. The mechanism of mitochondrial damage and autophagosome formation, which involves the sequential expansion of the phagophore, provides autophagy with the capacity to requisition essentially any cellular component and deliver it into the vacuole for degradation. Definitely, autophagy plays an important role in the degradation of cellular components, such as protein complexes, the endoplasmic reticulum, ribosomes, peroxisomes and mitochondria. Subsequently, autophagy plays an essential role in the digestion of damaged organelles within the cells and induces the secretion of damaged cell organelles in the form of exosomes [47, 48]. The graphical model shown in Figure 6, explains the molecular mechanism of association between mitochondrial damage, induction of autophagy and exosomes release in the rotenone treated CSCs. Results obtained from the exposure of CSCs to rotenone also gave us a clue to understand the mechanism of exosomes release and induction of autophagy in CSCs. Rotenone induces autophagic vacuolation in CSCs which are associated with the mitochondrial damage [3]. These processes induce the formation of autophagosomes and autophagy in CSCs [49–51]. Our results confirm that mitochondrial damage and induction of autophagy induces the formation and release of exosomes in rotenone treated prostate and breast CSCs. The carcinogenic effects of rotenone on male rats (F344/N) have also been reposted [52, 53].

Recently, the identification of exosomal RNA and protein components have been performed by the several researchers. The identification of various mRNA and microRNA within exosomes derived from the mouse and human mast cell lines [42], immature and mature dendritic cells [54] and human colorectal cancer cell lines have been reported [55]. These studies showed that RNA component within exosomes differed according to cell type. Herein, we examined the types of exosomal marker proteins (CD9, CD61 and CD81) and RNAs (CD9, CD61, CD81 and TSG101) within exosomes derived from the prostate and breast CSCs. These mRNAs and proteins may be used as biomarkers for prostate and breast cancer.

An understanding of exosome release and its regulation would be greatly enhanced by the identification of the proteins required. The link between cancer and multivesicular bodies was strengthened by the discovery of the association between autophagy and exosomes release from CSCs. The strategy was used in the lung cancer and melanoma trials, where, exosomes were harvested from tumor cells in abdominal fluid [22, 23]. Such cells and their exosomes contained antigens specific to the individual's cancer. In a study, when exosomes were administered along with a substance that stimulates the immune system, the vesicles seemingly trained the immune system to recognize cancer cells as foreign and attack the cells [22, 23], and the identification of active components of the conditioned medium as exosomes expressing the tetraspanin protein CD81. In a gene expression study, data set from human breast cancers showed that CD81 was significantly upregulated in the stroma associated with breast cancer compared with control breast tissue, suggesting that CD81 may also have a role in the cancer associated in human breast cancer [22, 23]. Interestingly, the releases of exosomes from prostate CSCs were higher than the exosomes released from the breast CSCs. The protein and mRNA expression of exosomal markers were significantly higher in exosomes released from prostate CSCs compare to breast CSCs.

In summary, we have demonstrated the biomolecular characterization of exosomes released from the rotenone treated prostate and breast CSCs. We found that rotenone induced the release of exosomes from prostate and breast CSCs, characterized by presence of several exosomal markers, CD9, CD63, CD81, Alix and TSG101. Alix is a multifunctional cytosolic protein, involved in several cellular processes, including endocytic membrane trafficking [56]. The induction of autophagy and release of exosomes from the CSCs suggest that the exosome release plays an important role in the formation of autophagosomes. The differentiation of blood cells involves the loss of organelles and specific proteins to allow the terminal differentiation in the mature erythrocyte, which is released into the peripheral blood and the best described systems to get rid of unnecessary molecules is the targeting of specific proteins to the internal MVB vesicles and their subsequent release into the extracellular medium through exosomes [57]. However, several organelles, such as mitochondria, Golgi apparatus and endoplasmic reticulum, also need to be eliminated. In this case, morphological evidence indicates that autophagy participates in the removal of these organelles [58]. We observed that the exosomes released from the rotenone treated prostate and breast CSCs expressed higher levels of CD9, CD63, CD81, Alix and TSG101. It indicates that the release of exosomal markers and oncogenes could be highly relevant for the biological activity, and can be used as a potential target for the exosomes mediated tumor development. Taken together, our data suggests that the constituents of exosomes can carry information from cell to cell (act as a cargo to deliver proteins and nucleic acids), and can be used as a biomarker for the targeted therapy of cancer.

## MATERIALS AND METHODS

### Cell culture and reagents

CSCs were obtained from human primary tumors as described previously [49, 59]. Cells were routinely grown in complete stem cells growth medium (Celprogen, San Pedro, CA) with 1% antibiotic-antimycotic (Invitrogen). Rotenone was obtained from Sigma-Aldrich Corp. (St. Louis, MO). Anti-human LC3, Atg7 and β-actin antibodies were purchased from Cell Signaling Technology (Danvers, MA). CD9, CD63, CD81 and Alix were purchased from eBioscience, Inc. (San Diego, CA).

### Exosomes isolation

CSCs were grown in complete stem cells growth medium (Celprogen, San Pedro, CA) with 1% antibiotic-antimycotic (Invitrogen). Media were collected after 48 h of exposure of CSCs with rotenone (40 μM). Media were centrifuged at 300×g for 10 min at 4°C to pellet the cell debris. Supernatant were filtered through 0.2 μm membrane filter to remove particles larger than 200 nm. After filtering supernatant were centrifuged at 120,000×g for 90 min at 4°C to pellet the exosomes. Supernatant were discarded and pellet were resuspended in RIPA buffer for protein analysis and in lysis buffer for RNA isolation.

### Electron microscopy

To demonstrate the mitochondrial damage in rotenone treated CSCs, cells were grown on 13 mm diameter coverslips and exposed with rotenone (0 and 40 μM) for 48 h. Cells were washed twice with PBS and fixed in 2.0% glutaraldehyde in 0.1 M phosphate buffer, then post-fixed in 1% osmium tetroxide buffer. After dehydration in a graded series of ethanol, cells were embedded in spur resin. Thin sections (60 nm) were cut on an ultramicrotome. The sectioned grids were stained with saturated solutions of uranyl acetate and lead citrate. The sections were examined under electron microscope.

### Fluorescence microscopy

CSCs were grown on fibronectin-coated coverslips (Beckton Dickinson, Bedford, MA), and exposed with rotenone (0 and 40 μM), washed in PBS, and fixed for 15 min in 2% paraformaldehyde. Cells were permeabilized in 0.1% Triton X-100, washed and blocked in 10% normal goat serum. After blocking, cells were incubated with alexa fluor tagged antibody against CD9, CD63, CD81 and Alix (1:100) overnight at 4°C. Cells were washed with PBS and incubated with 4, 6-diamido-2-phenylindole hydrochloride (DAPI) (1 mg/ml) for 1 h at room temperature. Finally, coverslips were washed and mounted using vectashield (Vector Laboratories, Burlington, CA). Isotype-specific negative controls were included with each staining. Stained cells were mounted and visualized under Leica 6000B microscope with 100X objectives. The number of cells expressing punctate and the number of punctate per cell were counted under fluorescence microscope.

### Western blotting

To allow the detection of autophagy related genes (LC3 and Atg7), exosomal markers (CD9, CD63 and CD81) and β-actin, the exosomes from rotenone (0 and 40 μM) treated CSCs were digested with RIPA buffer (50 mM Tris-HCl, pH 7.5, 150 mM NaCl, 1% v/v Nonidet P-40, 0.5% v/v sodium deoxycholate and 0.1% SDS) supplemented with protease inhibitor cocktail (Sigma) and phosphatase inhibitor cocktail (Sigma), and lysed on ice by sonication for 2 s and 2 pulses. The lysates were centrifuged for 20 min at 12,000xg, and supernatant was collected and used for further experiments. Equal amount of protein lysates (50–60 μg total protein) were electrophoretically separated by 10% and 15% sodium dodecyl sulfate-polyacrylamide gel electrophoresis (SDS-PAGE) and transferred to nitrocellulose membrane. Nitrocellulose blots were blocked with 5% nonfat dry milk in TBS-T (TBS and 0.01% Tween-20) buffer (20 mM Tris-HCl (pH 7.4), and 500 mM NaCl), and incubated with primary antibody (1:1000) in TBS-T overnight at 4°C. Immunoblots were washed three times (5, 5 and 5 min each) with TBS-T followed by incubation with HRP tagged secondary antibody (1:10000). Chemiluminescence reactions were carried out according to the Super Signal West Pico substrate (Thermo Fisher, Waltham, MA) protocol. Antibody dilutions were carried out as per the data sheet provided by the manufacturer. Blots were stripped for reuse by washing for 30 min to 2 h in TBS-T buffer (pH 2.0–2.5) at room temperature.

### Exosomal RNA isolation and expression of mRNAs by qRT-PCR

CSCs were grown in complete stem cell growth medium (Celprogen, San Pedro, CA) with 1% antibiotic-antimycotic (Invitrogen). Media were collected after 48 h treatment of CSCs with rotenone (0 and 40 μM). Media were centrifuged at 300×g for 10 min at 4°C to pellet the cell debris. Supernatant were filtered through 0.2 μm membrane filter to remove particles larger than 200 nm. After filtering supernatant were centrifuged at 120,000×g for 90 min at 4°C to pellet the exosomes. Supernatant were discarded and pellets were lysed in TRI reagent (ambion). Total RNAs were isolated using the TRI reagent protocol, and synthesis of cDNAs was performed by oligo (dT)-priming methods. qRT-PCR was performed using SYBR Green Master Mixes (life technology) according to the manufacturer's instructions. Primers specific for each of the signaling molecules were designed using the National Center of Biotechnology Information/Primer-Basic Local Alignment Search Tool and were used to generate the PCR products. Expression levels of glyceraldehyde-3-phosphate dehydrogenase (GAPDH) were used for normalization and quantification of gene expression levels. For the quantification of gene amplification, qRT-PCR was performed using an Applied Biosystems 7300 Sequence Detection System in the presence of SYBR Green. The following gene-specific primers were used:

Human CD63
Forward Primer 5′-ATG ATC ACG TTT GCC ATC TT-3′
Reverse Primer5′-AGG GAT TTT CTC CCA ATC TG-3′
Human CD9
Forward Primer 5′-TCT TGG TGA TAT TCG CCA TT-3′
Reverse Primer 5′-TTC GAG TAC GTC CTT CTT GG-3′
Human CD81
Forward Primer 5′-CTG TAT CTG GAG CTG GGA GA-3′
Reverse Primer 5′-GAA CTG CTT CAC ATC CTT GG-3′
Human TSG101-SET-1
Forward Primer 5′-CTC TCA TCT CTG CGG TCA GT-3′
Reverse Primer 5′-TCA ACC TCG GCT ACT TCT TG-3′
Human TSG101-SET-2
Forward Primer 5′-ATG CCT ATG GCT ACT GGA CA-3′
Reverse Primer 5′-GTC TGA CTG TGG GTC TTT CC-3′
Housekeeping-GAPDH
Forward Primer 5′-GAG TCA ACG GAT TTG GTC GT-3′
Reverse Primer 5′-TTG ATT TTG GAG GGA TCT CG-3′

### Statistical analysis

P-values were calculated by two-tailed t-test. Experiments were conducted thrice independently in triplicate. Data are shown as means ± s.e.m of values obtained from the experiments.

## Abbreviations

Atg: Autophagy related gene
CSC: Cancer Stem Cell
DAPI: 4′,6-diamidino-2-phenylindole
FBS: Fetal bovine serum
LC3: Microtubule-associated protein 1 light chain 3
miRNA: micro RNA
